# Beyond the pandemic: tracing the evolution of activity, screen time, and sleep in European children over 3 years

**DOI:** 10.1007/s00431-025-06458-1

**Published:** 2025-09-23

**Authors:** Mireia Orgilés, Víctor Amorós-Reche, Rita Francisco, Cristina Godinho, Elisa Delvecchio, Claudia Mazzeschi, Marta Pedro, Alexandra Morales, Jose P. Espada

**Affiliations:** 1 Centro de Investigación de la Infancia y la Adolescencia, Universidad Miguel Hernández, Elche, Spain; 2 Católica Research Centre for Psychological, Family and Social Wellbeing, Universidade Católica Portuguesa, Lisbon, Portugal; 3 National School of Public Health, Public Health Research Center, Comprehensive Health Research Center, NOVA University, Lisbon, Portugal; 4 Department of Philosophy, Social Sciences and Education, Università Degli Studi Di Perugia, Perugia, Italy

**Keywords:** Adolescents, Children, COVID-19 pandemic, Digital devices, Exercise, Sleep behavior

## Abstract

During COVID-19, several studies documented a decrease in physical activity time, an increase in screen use and a worsening of sleep duration. The aim of this study was to compare the proportion of children with unhealthy amounts of time dedicated to these three habits across three different moments: before the pandemic (T1), 2 weeks after its outbreak (T2), and three and a half years later (T3), when the situation was fully restored. A total of 1248 caregivers of children and adolescents aged 3 to 18 years old (46.9% female) from Italy, Spain and Portugal reported the amount of time devoted to physical activity, screen use and sleep at each moment. At T2, an increase in the percentage of children and adolescents with unhealthy time dedicated to physical activity and screen use was recorded. Proportions decreased at T3 but remained higher than at T1. At T3, the proportion of participants with inadequate sleep hours significantly decreased in children aged 3 to 5 compared to T1–T2, showed no differences in children aged 6 to 12, and increased in adolescents compared to T2, with no significant differences compared to T1.

*Conclusion*: Results highlight that, although unhealthy patterns in physical activity and screen use have decreased compared to the confinement in March 2020, three and a half years later they remain higher than before the COVID-19 pandemic. These findings underscore the need for continued efforts to promote healthy lifestyles and prevent potential adverse consequences.

**Table:** 

What is known? • *The COVID-19 pandemic disrupted children’s and adolescents’ routines, leading to impairments in physical activity, screen use, and sleep*. • *Some of these difficulties persisted throughout the pandemic, even without confinement measures, while others gradually improved*.
What is new? • *Three and a half years after the start of the pandemic, the proportion of children and adolescents with unhealthy durations in physical activity and screen use had decreased compared to during the confinement, but remained significantly higher than before the pandemic*. • *Sleep duration does not significantly differ -or even improves, depending on the age group- relative to before the pandemic*.

What is known?

• *The COVID-19 pandemic disrupted children’s and adolescents’ routines, leading to impairments in physical activity, screen use, and sleep*.

• *Some of these difficulties persisted throughout the pandemic, even without confinement measures, while others gradually improved*.

What is new?

• *Three and a half years after the start of the pandemic, the proportion of children and adolescents with unhealthy durations in physical activity and screen use had decreased compared to during the confinement, but remained significantly higher than before the pandemic*.

• *Sleep duration does not significantly differ -or even improves, depending on the age group- relative to before the pandemic*.

The outbreak of the COVID-19 pandemic had a significant impact on the lives of children and adolescents, leading to an unprecedented situation. On March 9th and 14th, 2020, the Italian and Spanish governments, respectively, implemented mandatory home confinement, which was only recommended in Portugal, although with schools closed nationwide. The confinement and restrictions imposed to mitigate contagion resulted in increased time at home and a disruption of routines, characterized by reduced physical activity, increased screen time, and inadequate sleep duration [20]. Prior to the pandemic, evidence suggested that periods without school attendance and the absence of routines contribute to an increase in these unhealthy behaviors [3].

Different studies have revealed a general increase in sedentary lifestyles and a decrease in physical activity among children and adolescents with the onset of the pandemic [7]. The meta-analysis by Ludwig-Walz et al. [17] indicated a reduction of 48 min in total daily physical activity, as well as a 12-min reduction in moderate-vigorous physical activity. Another meta-analysis [5] found that the prevalence of physical activity in children decreased from 46.4% before the pandemic to 19.5% during restrictions. The reduction and non-compliance with the guidelines proposed by the World Health Organization (WHO) were greater in children than in adults. These results suggest a greater vulnerability of children in these circumstances, as less physical activity during the pandemic has been related to greater anxiety and depressive symptoms [15]. With the easing of lockdown restrictions, an increase in physical activity among children has been observed [14, 16]. However, qualitative research has highlighted a trend toward a reduction in spontaneous physical activity even with the return to schools during the pandemic [26]. Similarly, 1 year after the start of the pandemic, Moore et al. [19] found reductions in the sedentary behavior of children and adolescents, although with no changes in engagement in physical activity. However, following a wider period from late 2020 to late 2022 in Spanish children, Portals-Riomao et al. [22] identified an upward trend in meeting recommendations.

Regarding the use of screens during the pandemic in children and adolescents, a meta-analysis of 91 studies showed an increase from 2.67 to 4.38 h per day on average [6]. The increase was especially notable in boys compared to girls and older children compared to younger children. However, in children under 5 years of age, there was also an increase of almost an hour, with daily screen time exceeding two and a half hours. One of the main reasons for the increased screen use in children and adolescents was the shift in education toward a virtual or hybrid format [10, 24]. According to Moavero et al. [18], 72% of children increased their screen use for educational purposes and 50% for recreational purposes. Hedderson et al. [11] reported that for both purposes, daily use increased by almost an hour. A year after the lockdown, total usage was still increased compared to pre-pandemic data, with more than an additional hour. Only slight improvements or no significant differences in screen use were found between the end of 2020 and the end of 2022 [22]. Therefore, screen-viewing activities may have gained prominence at the expense of other social activities and are seen as a residual habit of the pandemic [26].

Associations have been found between increased screen use and poorer sleep, both during the pandemic [18, 24] and previously [12]. With studies conducted at various points during the pandemic, the meta-analysis by Cai et al. [4] reflects a prevalence of sleep disturbances in 42.2% of children and 21.1% of adolescents. According to Moavero et al. [18], the pandemic led to a 12-point increase in the percentage of pre-pandemic sleep disturbances. During confinements, children went to bed and woke up later, according to Tatsiopoulou et al. [24], Hassinger et al. [10], and Kaditis et al. [13]. Of these three studies, the first reflected that more than 60% of children under 6 years of age had a problematically reduced sleep duration. In the second, conducted with children aged 5 to 13 years, sleep time increased during confinement. However, with the reopening of schools, this time decreased, showing no differences compared to pre-pandemic data. The third study reflected that adolescents aged 14 to 17 had a longer sleep duration compared to younger children during the lockdown.

Throughout the COVID-19 pandemic, several changes in the lifestyles of children and adolescents have been observed. A previous longitudinal study conducted with Italian, Spanish, and Portuguese children and adolescents aged 3–18, during various time points of the confinement, showed the progression of some habits. The proportion of children with unhealthy physical activity duration increased 2 weeks after the start of the confinement. Despite a slight decrease as the lockdown progressed, after 8 weeks, it remained significantly higher than before the pandemic. The use of screens increased with the start of the lockdown and was maintained over the weeks, with some variations according to the age range. Furthermore, there were no significant changes in children and adolescents with an unhealthy amount of sleep hours from before the pandemic to 2 weeks after its start, but this proportion increased 5 weeks after and decreased 8 weeks after [20]. Some studies suggest that changes in lifestyles have been maintained over time, even with the lifting of restrictions [11, 19, 22]. However, no study has yet been published to determine whether these changes in habits are maintained once the situation has normalized and restrictions have been lifted. Continuing with the mentioned previous study [20], the aim of this study was to compare healthy and unhealthy patterns of activity, screen time, and sleep in children from three European countries across three different periods: before the pandemic, during lockdown, and three and a half years after lockdown.

## Materials and methods

### Participants

The study involved 1248 caregivers of European children and adolescents within the age range of 3 to 18 years (mean age = 10.15, *SD* = 4.38). The sample of youth was almost evenly divided by gender, with 46.9% being female. Participants were recruited from Italy (*n* = 769), Spain (*n* = 235), and Portugal (*n* = 244). The caregivers were majorly female (88.4%), with a significant proportion being married (86.5%). The average age of the caregivers was 43.32 years (*SD* = 6.08). More information about the characteristics of the sample is described in Table 1.

**Table 1:** Sample characteristics

	(*N* = 1248)
**Parents**	
Female, *n* (%)	1103 (88.4)
Age, *M* (*SD*)	43.32 (6.08)
Country, *n* (%)	
Italy	769 (61.6)
Spain	235 (18.8)
Portugal	244 (19.6)
Marital status, *n* (%)	
Married	1079 (86.5)
Single	160 (12.8)
Other	9 (0.7)
Educational level, *n* (%)	
Doctoral or master	305 (24.4)
Undergraduate	534 (42.8)
Secondary school	356 (28.5)
Primary school	53 (4.2)
Monthly family income (euros), *n* (%)	
Up to 999	47 (4.3)
Between 1000 and 1999	301 (27.3)
Between 2000 and 2999	330 (29.9)
Between 3000 and 4999	291 (26.4)
5000 or more	101 (9.1)
I prefer do not inform	24 (2.7)
**Children**	
Female, *n* (%)	585 (46.9)
Age, *M* (*SD*)	10.15 (4.38)

*M =* mean, *SD* = standard deviation

### Procedure

This study is part of a larger longitudinal study to monitor the evolution of psychological symptoms and habits in children and adolescents across different times of the COVID-19 pandemic. The study received ethical approval from the Ethics Committee of Miguel Hernández University of Elche, guaranteeing compliance with the highest standards of research integrity and the welfare of participants.

This longitudinal study was conducted during the COVID-19 pandemic in Italy, Spain, and Portugal, using an online survey designed on Google Forms as the principal method for data collection. Prior to participation at any timepoint, parents received detailed study information and provided informed consent through a mandatory checkbox before accessing the questionnaire. To link responses across waves, parents were instructed to create a self-generated identification code based on a combination of letters and numbers. The resulting dataset was stored according to the EU General Data Protection Regulation. The snowball sampling strategy for participant recruitment was used through social media platforms (including Instagram and Facebook), email and WhatsApp. A meticulous protocol ensured consistent data collection across countries. Participation was restricted to one child per parent or legal guardian to maintain the integrity and reliability of the dataset. Data were collected at two timepoints. In March 2020, T1 was assessed a retrospective measurement of the situation before confinement, as well as T2, referred to the current situation 2 weeks after the onset of the pandemic. At that time, schools were closed in all participating countries, with children in Italy and Spain undergoing continuous mandatory home confinement, which was only recommended in Portugal. The T3 assessment was conducted in September 2023, when normalcy had resumed three and a half years after the onset of the pandemic.

### Measures

The assessment tools were culturally tailored for each of the three countries and underwent a pilot study to verify item comprehensibility. Parents of the child participants filled out the following assessments online.

A general sociodemographic questionnaire was used, where caregivers provided information about their age, sex, marital status, level of education, monthly family income, as well as their child’s age and sex.

Physical activity levels were determined by initially asking caregivers about their child’s daily physical activity duration before the quarantine period. Furthermore, at every assessment point, parents reported the amount of time their children dedicated to physical activity. The response scale included the following alternatives: (1) Less than 30 min, (2) 30 to 60 min, (3) 60 to 90 min, (4) 90 to 120 min, (5) 120 to 180 min, and (6) More than 180 min. According to WHO guidelines [27, 28], a minimum of 60 min of physical activity per day is recommended for children and adolescents. Thus, physical activity responses were categorized into unhealthy pattern (less than 60 min daily) and healthy pattern (more than 60 min daily).

Screen time was assessed by inquiring how long before the quarantine children and adolescents were exposed to screens daily, including iPads, televisions, smartphones, or computers. This question was repeated at each subsequent assessment. The options provided were similar to those for physical activity, with (1) Less than 30 min, (2) 30 to 60 min, (3) 60 to 90 min, (4) 90 to 120 min, (5) 120 to 180 min, and (6) More than 180 min. Following age-specific recommendations from pediatric guidelines [1, 2, 25], screen time was considered excessive when it exceeded 60 min per day for children aged 3–11, 90 min for those aged 12–15, and 120 min for adolescents aged 16–18. These thresholds allowed for a developmentally informed categorization of screen use, in line with health authorities’ emphasis on minimizing sedentary behavior while adapting limits to age and autonomy.

Sleep duration was established by asking caregivers about their child’s weekly sleep hours before, during, and after COVID-19 quarantine. Responses were given in numerical form. The American Academy of Pediatrics advises that children aged 3–5 should get 10 to 13 h of sleep, those aged 6–12 should sleep for 9 to 12 h, and teenagers aged 13–18 should have 8 to 10 h of sleep [21]. This served as the basis for categorizing sleep hours into adequate (healthy group) and inadequate (unhealthy group) sleep durations.

Before full data collection, a brief pilot study was conducted in each participating country to evaluate the clarity and cultural adequacy of the items. A small group of caregivers (*n* ≈ 10 per country) completed the initial version of the survey and provided feedback on the comprehensibility of the items. No major changes to item content were necessary, suggesting satisfactory validity.

### Data analysis

Descriptive statistical analysis provided a detailed characterization of the sample, including means and standard deviations for quantitative variables, alongside frequencies and percentages for categorical variables. An attrition analysis was conducted to delineate the attributes of participants who ceased participation in the study. Utilizing logistic regression, differences in the main outcomes between participants who concluded the study (denoted as “1”) and those who discontinued (denoted as “0”) were analyzed.

To study the evolution of physical activity duration, screen exposure, and sleep patterns across the three assessments, repeated measure data analysis was executed employing generalized estimating equation (GEE) models. Each behavioral domain was analyzed independently through models that integrated a selection of variables. This approach adhered to the principle of parsimony and considered the necessity of multiple comparisons. Incorporated variables in each model comprised the primary outcome, a temporal variable for cross-time comparisons, and key sociodemographic factors –specifically, the child’s age and gender, along with the baseline measurement of the outcome (T1). A *p*-value below 0.05 was indicative of statistically significant differences. The online survey platform mitigated the issue of missing data by prompting participants to complete any missed items, ensuring comprehensive data collection. The data were analyzed utilizing IBM SPSS for Windows, Version 28.

## Results

### Attrition

A total of 1248 participants completed the T1 and T2 assessment simultaneously in March 2020. The retention rate for T3 was 37.1% (*n* = 463). Participants did not provide reasons for discontinuing their participation in the study. The retention rate at T3 was notably higher in Spain (55.7%) and Portugal (65.6%), compared to Italy (22.4%) (*p* < 0.05). Participants who did not respond at T3 were found to be comparable to those who remained in the study, except for differences in screen time usage and hours of sleep. In T3, a significantly larger portion of participants previously categorized under “healthy screen use” in the age group of 3 to 5 years participated, in comparison to the “unhealthy screen use” group (65.2% vs 34.8%, *OR* = 1.85, 95% CI: 1.13, 3.04, *p* = 0.04). Furthermore, a greater percentage of participants reporting that their children aged 3 to 5 years exhibited an unhealthy sleep pattern participated in T3, in contrast to the “healthy sleep pattern” group (55.9% vs 44.1%; *OR* = 2.40, 95% CI: 1.46, 3.94, *p* = 0.001).

### Lifestyle habits during home confinement

**Fig. 1:**
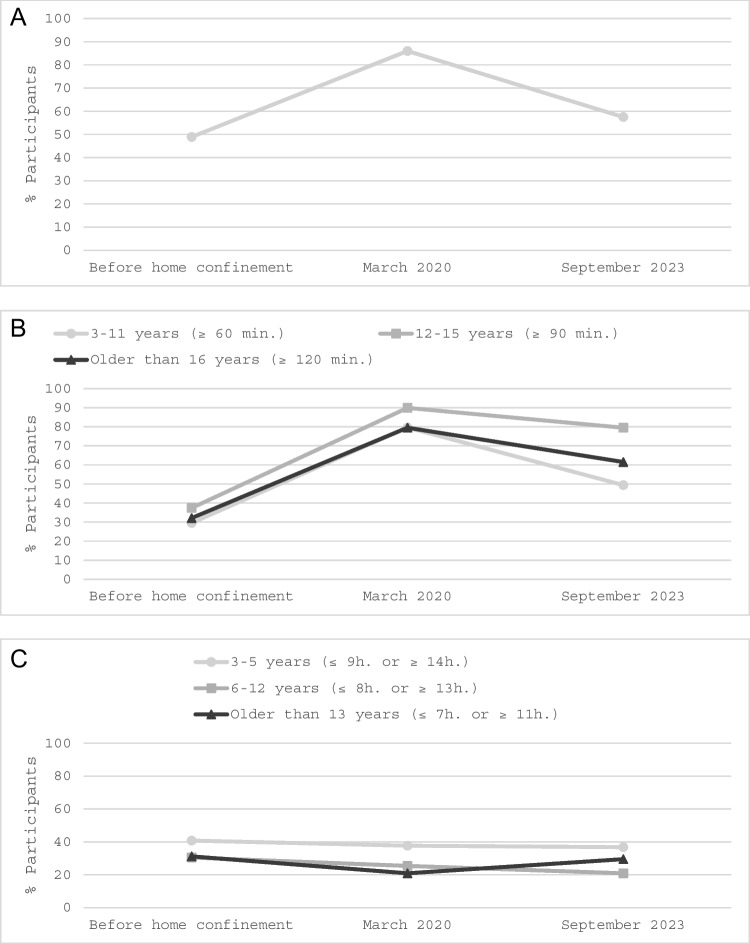
Percentage of children and adolescents not meeting guidelines for physical activity (**A**), screen time (**B**), and sleep duration (**C**)

Table 2 and Fig. 1 showcase the proportion of children categorized within the unhealthy groups based on daily physical activity (less than 60 min), screen time (exceeding age-recommended limits), and sleep duration (deviating from the recommended hours) over three distinct periods: a retrospective evaluation of habits prior to confinement (T1), 2 weeks into the lockdown (T2), and three and a half years after the start of the pandemic (T3). The comparisons of T3 against T1 and T2 are facilitated using GEE models (Table 3).

**Table 2:** Percentages of child lifetime habits at the three time points

Physical activity		Use of screens			Sleep hours		
	3–18 years	3–11 years	12–15 years	16 years and older	3–5 years	6–12 years	13 years and older
	≤ 60 min	≥ 60 min	≥ 90 min	≥ 120 min	≤ 9 h or ≥ 14 h	≤ 8 h or ≥ 13 h	≤ 7 h or ≥ 11 h
	*n* (%)	*n* (%)	*n* (%)	*n* (%)	*n* (%)	*n* (%)	*n* (%)
T1	495/1012 (48.9)	207/702 (29.5)	74/198 (37.4)	36/112 (32.1)	113/277 (40.8)	147/482 (30.5)	79/253 (31.2)
T2	870/1012 (86.0)	559/702 (79.6)	178/198 (89.9)	89/112 (79.5)	103/273 (37.7)	122/481 (25.4)	53/253 (20.9)
T3	266/463 (57.5)	132/267 (49.4)	99/144 (68.8)	32/52 (61.5)	7/19 (36.8)	58/278 (20.9)	49/166 (29.5)

*T1 =* retrospective measurement of before home confinement, *T2* = during the first 2 weeks of lockdown (March 2020), *T3* = three and a half years after the start of the pandemic (September 2023). % = Number of cases that do not meet the healthy guidelines / total of cases available for analysis

**Table 3:** Evolution of children and adolescents’ lifestyle: results from GEE models

	Physical activity		Use of screens						Sleep hours					
	3–18 years		3–11 years		12–15 years		16–18 years		3–5 years		6–12 years		13–18 years	
	Estimates (95% CI)	*p*	Estimates (95% CI)	*p *	Estimates (95% CI)	*p*	Estimates (95% CI)	*p*	Estimates (95% CI)	*p*	Estimates (95% CI)	*p*	Estimates (95% CI)	*p*
**Time**														
T1	2.67 (1.34, 5.29)	.005	7.78 (4.35, 13.90)	<.001	0.004 (0, 0.041)	<.001	0.04 (0.009, 0.22)	<.001	0.24 (0.14, 0.40)	<.001	0.86 (0.33, 2.24)	.76	0.63 (0.29, 1.36)	.24
T2	0.12 (0.068, 0.22)	<.001	0.06 (0.02, 0.16)	<.001	2.42 (1.24, 4.74)	.01	3.35 (1.11, 10.11)	.03	0.06 (0.03, 0.12)	<.001	0.45 (0.16, 1.27)	.13	0.33 (0.12, 0.90)	.03
T3	-	-	-	-	-	-	-	-	-	-	-	-	-	-

Estimates from the GEE model predicting differences in the proportion of participants meeting the guidelines between T3 and T1/T2. *T1* = retrospective measurement of before home confinement; *T2* = during the first 2 weeks of lockdown (March 2020); *T3* = three and a half years after the start of the pandemic (September 2023). Models adjusted for the child’s age, gender and baseline value of the outcome (T1)

#### Physical activity

Across all ages, there was a notable decline in physical activity during the initial lockdown, with an increase in children engaging in less than 60 min of daily physical activity from T1 (48.9%) to T2 (86%). Although there was a significant reduction in this trend by T3 (57.5%) compared to T2, the levels did not fully revert to pre-pandemic norms, being significantly higher than at T1.

#### Screen usage

For adolescents aged 12–15, there was an alarming rise in screen time usage exceeding the recommended limit, escalating from 37.4% at T1 to 89.9% at T2. By T3, an important proportion (68.8%) remained above the recommended screen time usage, with significantly lower levels than at T2, but higher than at T1. Children aged 3–11 and adolescents aged 16 and older demonstrated similar patterns, with an increase from T1 to T2 and sustained elevated levels of screen usage at T3, but significantly lower than during the pandemic.

#### Sleep patterns

The patterns of sleep adherence shifted across different age groups. The data suggests that the proportion of the youngest children (aged 3–5 years) who exhibited unhealthy sleep patterns was lower at T3 (36.8%), compared to T2 (37.7%) and T1 (40.8%), with a decrease over time. For the 6- to 12-year-old group, although a reduction was also identified, no statistically significant changes were found. For the 13- to 18-year-old group, a significant increase in the proportion of adolescents with unhealthy sleep habits was registered from T2 (20.9%) to T3 (29.5%), with no differences in T3 compared to T1 (31.2%).

## Discussion

The aim of this study was to compare the proportion of children with healthy and unhealthy habits concerning activity, screen time, and sleep in European children in three moments: before and during the lockdown and when the situation was restored three and a half years after, in September 2023.

First, it was found that, before the pandemic, almost half of the participating children and adolescents did not comply with the WHO guidelines of 60 min of physical activity per day. Two weeks after the confinement, this percentage rose remarkably, following the bulk of the scientific literature [5, 7, 17] and revealing that more time at home and movement restrictions made it difficult for children to practice physical activity and meet the recommended minimum time. Three and a half years after the confinement, the proportion of children and adolescents with unhealthy physical habits decreased, also following the evidence [14, 16, 22]. However, compared to the retrospective measure prior to the pandemic, it had increased by almost 10 points, consistent with other studies suggesting a residual lack of habits in terms of physical activity, despite the lifting of all restrictions [19, 26]. These findings would indicate a lasting impact of the pandemic on physical activity of children and adolescents.

During the lockdown, the proportion of children with problematic screen use increased by about 50 points in all age groups. These results align with the average increase of 1.71 hours in front of the screen reflected by the meta-analysis by Choi et al. [6], an increase that may cross unhealthy limits. More time at home with fewer leisure opportunities, as well as the shift toward an online education format [10, 11, 18, 24] may have been responsible for this elevation in unhealthy screen habits. The excessive screen use especially affected the group of adolescents between 12 and 15 years of age, with the healthy limit exceeded by 89.9% of the participants, following previous evidence that showed a higher screen use in older children [6]. Compared to 2 weeks after the start of the lockdown, three and a half years later, a significant reduction was observed in the percentages of children and adolescents who used screens excessively [22]. However, in all age groups, the proportions were still significantly higher than before the lockdown. This finding adds to others that indicate that the greater presence of technological media and online activities during the pandemic seems to have been a turning point in the increase of screen use by children and adolescents [11, 26].

Regarding excess or deficit of sleep hours, 2 weeks after the start of the lockdown, a slight decrease in the proportion of all participants with an unhealthy sleep duration was observed. In children aged 3 to 5 years, the proportion of participants who slept an inadequate number of hours was lower than that reported by Tatsiopoulou et al. [24] during a new lockdown 1 year later. Hassinger et al. [10] found that, during confinement, children slept on average 25 min longer. Despite the disruption of sleep schedules observed during confinement [10, 13, 24], this period may have allowed sleep hours to fall within healthy limits. Three and a half years later, the proportion of children aged 3 to 5 with unhealthy sleep hours was significantly lower than 2 weeks after the start of the pandemic, but also before its onset. Children aged 6 to 12 years followed a similar pattern, although with no significant differences between the most recent time and the other two periods, consistent with Portals-Riomao et al. [22]. Our findings suggest that the pandemic may not have negatively affected sleep duration and might even have contributed to establishing healthier sleep patterns in children up to 12 years old. Adolescents aged 13 and older followed a slightly different pattern. During the lockdown, there was a decrease in the percentage of adolescents with unhealthy sleep duration. According to literature, in this period, adolescents presented a greater increase in sleep hours [13] and had a lower prevalence of sleep problems compared to children [4]. However, three and a half years later, a significant increase was observed in the percentage of adolescents with unhealthy sleep hours compared to confinement, with no significant differences compared to before the pandemic.

Several practical implications may arise from these findings. First, the impact of confinement on lifestyle, characterized by decreased physical activity and increased screen use, persisted over time. Despite trends toward a return to “normalcy,” consistent with prior literature [22], the pandemic appears to have had a lasting effect on how time is allocated to these activities. Given that physical activity during childhood and adolescence is associated with better mental health outcomes [23] and that certain forms of screen use are linked to poorer mental health [29], the prevention of unhealthy behaviors should be a public health priority, which includes promoting alternative and screen-free activities. Second, while significant reductions or no significant differences in the proportion of children with an unhealthy sleep duration were found, adolescents showed a slight worsening in sleep patterns compared to 2 weeks after the onset of the pandemic. Previous studies have associated increased screen time, particularly after 6 p.m., with poorer sleep quality [18]. Since behavioral interventions have been shown meta-analytically to improve sleep duration in adolescents and young adults without clinical sleep disorders [9], implementing such strategies could help mitigate the negative effects of screen use on sleep.

This study is not without limitations. First, the attrition in the third assessment (September 2023) was high. Additionally, the constructs examined in the present study were assessed using ad hoc items rather than validated questionnaires, and the first assessment was a retrospective measure. Inconsistencies have been observed between measures obtained previously and retrospective measures during confinement [8], so the new situation and uncertainty may have altered parents’ perceptions. Although parent-reported data may be subject to recall bias and subjectivity, we aimed to minimize this effect by using clear, simple, and time-based questions (e.g., hours per day) and collecting data during a period when parents were closely involved in their children’s routines, at least in the first evaluation. In addition, physical activity was not categorized by intensity (i.e., light, moderate or vigorous), nor was it specified whether it involved organized sports, active play or other types of activity. Non-probabilistic sampling through social networks and parent-reported evaluation—especially for adolescents—which had to be carried out in this way due to the circumstances of the pandemic, could be considered other limitations. Moreover, it would have been valuable to include assessments at intermediate time points, allowing comparisons between the confinement and other moments of the pandemic, such as the reopening of schools in September 2020. Finally, stratified analyses by sex, age group, or country were beyond the scope of the current work and would have substantially reduced the statistical power of the analyses, especially considering the reduced sample size in the third wave due to attrition. Future studies with larger and more balanced samples are encouraged to explore potential differences across these sociodemographic variables. Nevertheless, this study has made it possible to compare children’s lifestyles before, during, and after the pandemic, when the situation was fully restored. Furthermore, it includes a large sample from three European countries: Italy, Spain and Portugal. To the best of our knowledge, this is the first study to include in comparisons of healthy lifestyles in children a time point as recent as September 2023. Thus, as a part of a larger study, this work adds understanding to the evolution of habits in children, not only during the COVID-19 confinement [20], but also after the pandemic.

In conclusion, 2 weeks after the lockdown, there was an increase in the proportion of children and adolescents with unhealthy habits of physical activity and screen use. Three and a half years later, in September 2023, the proportion of children with these unhealthy habits decreased but remained higher than before the pandemic. On the other hand, during confinement, children and adolescents seemed to improve their sleep duration. In September 2023, results varied according to age, with more adolescents aged 13 to 18 with inadequate sleep hours compared to confinement and the return to pre-pandemic levels. These results show that, despite a normalized situation after the pandemic without restrictions or alterations to routines, COVID-19 has brought changes in the daily lifestyle of children and adolescents. These patterns highlight the need for public health strategies that promote healthy lifestyles, particularly physical activity and screen-free routines, as well as interventions to support adequate sleep in adolescents.

## Data Availability

The data that support the findings of this study are not openly available.

## References

[1] American Academy of Pediatrics (2016) Media and young minds. Pediatrics 138(5):e20162591. 10.1542/peds.2016-2591 27940793 10.1542/peds.2016-2591

[2] Asociación Española de Pediatría (2024) La AEP actualiza sus recomendaciones sobre el uso de pantallas en la infancia y adolescencia [Press note]. https://www.aeped.es/noticias/aep-actualiza-sus-recomendaciones-sobre-uso-pantallas-en-infancia-y-adolescencia. Accessed 11 Sept 2025

[3] Brazendale K, Beets MW, Weaver RG, Pate RR, Turner-McGrievy GM, Kaczynski AT, Chandler JL, Bohnert A, von Hippel PT (2017) Understanding differences between summer vs. school obesogenic behaviors of children: the structured days hypothesis. Int J Behav Nutr Phys Act 14:100 28747186 10.1186/s12966-017-0555-2 PMC5530518

[4] Cai H, Chen P, Jin Y, Zhang Q, Cheung T, Ng CH, Xiang Y, Feng Y (2024) Prevalence of sleep disturbances in children and adolescents during COVID-19 pandemic: a meta-analysis and systematic review of epidemiological surveys. Transl Psychiatry 14:12.10.1038/s41398-023-02654-5 10.1038/s41398-023-02654-5 PMC10774396 38191533

[5] Chaabna K, Chaabane S, Jithesh A, Doraiswamy S, Mamtani R, Cheema S (2022) Effect of the COVID-19 pandemic on the proportion of physically active children and adults worldwide: a systematic review and meta-analysis. Front Public Health 10:1009703 36568744 10.3389/fpubh.2022.1009703 PMC9780669

[6] Choi EJ, King GKC, Duerden EG (2023) Screen time in children and youth during the pandemic: a systematic review and meta-analysis. Glob Pediatr 6:100080. 10.1016/j.gpeds.2023.100080

[7] Do B, Kirkland C, Besenyi GM, Smock C, Lanza K (2022) Youth physical activity and the COVID-19 pandemic: a systematic review. Prev Med Rep 29:101959 36034528 10.1016/j.pmedr.2022.101959 PMC9394097

[8] Dupuis M, Studer J, Wicki M, Marmet S, Gmel G (2023) Was retrospective change measurement conducted with Covid-19 containment inconsistent? Comparing prospective and retrospective change measures using data from a national survey on substance use and addictive behaviors. PLoS ONE 18(6):0286597 10.1371/journal.pone.0286597 PMC10237494 37267260

[9] Griggs S, Conley S, Batten J, Grey M (2020) A systematic review and meta-analysis of behavioral sleep interventions for adolescents and emerging adults. Sleep Med Rev 54:101356. 10.1016/j.smrv.2020.101356 32731152 10.1016/j.smrv.2020.101356 PMC7669566

[10] Hassinger AB, Monegro A, Perez G (2023) Parental survey of the sleep patterns and screen time in US school children during the first 6 months of the COVID-19 pandemic. BMC Pediatr 23:65 36750939 10.1186/s12887-023-03875-9 PMC9905756

[11] Hedderson MM, Bekelman TA, Li M, Knapp EA, Palmore M, Dong Y, Elliott AJ, Friedman C, Galarce M, Gilbert-Diamond D, Glueck D, Hockett CW, Lucchini M, McDonald J, Sauder K, Zhu Y, Karagas MR, Dabelea D, Ferrara A (2023) Trends in screen time use among children during the COVID-19 pandemic, July 2019 through August 2021. JAMA Netw Open 6(2):2256157 10.1001/jamanetworkopen.2022.56157 PMC9932850 36790805

[12] Janssen X, Martin A, Hughes AR, Hill CM, Kotronoulas G, Hesketh KR (2020) Associations of screen time, sedentary time and physical activity with sleep in under 5s: a systematic review and meta-analysis. Sleep Med Rev 49:101226 31778942 10.1016/j.smrv.2019.101226 PMC7034412

[13] Kaditis AG, Ohler A, Gileles-Hillel A, Choshen-Hillel S, Gozal D, Bruni O, Aydinoz S, Cortese R, Kheirandish-Gozal L (2021) Effects of the COVID-19 lockdown on sleep duration in children and adolescents: a survey across different continents. Pediatr Pulmonol 56(7):2265–2273 33887116 10.1002/ppul.25367 PMC8251495

[14] Kopp PM, Möhler E, Gröpel P (2024) Physical activity and mental health in school-aged children: a prospective two-wave study during the easing of the COVID-19 restrictions. Child Adolesc Psychiatry Mental Health 18:4. 10.1186/s13034-023-00695-8 10.1186/s13034-023-00695-8 PMC10765890 38172986

[15] Li B, Ng K, Tong X, Zhou X, Ye J, Yu JJ (2023) Physical activity and mental health in children and youth during COVID-19: a systematic review and meta-analysis. Child Adolesc Psychiatry Ment Health 17:92 37468975 10.1186/s13034-023-00629-4 PMC10357657

[16] Li Y, Zhao G, Su L, Fu J, Sun S, Chen R, Chen D, Hu X, Jiang T, Shen F (2024) The “supercompensation” effect of children’s lockdown during COVID-19: based on the analysis of changes in physical activity, sleep, and psychology. BMC Public Health 24:1522 38844937 10.1186/s12889-024-19035-2 PMC11154994

[17] Ludwig-Walz H, Siemens W, Heinisch S, Dannheim I, Loss J, Bujard M (2023) How the COVID-19 pandemic and related school closures reduce physical activity among children and adolescents in the WHO European Region: a systematic review and meta-analysis. Int J Behav Nutr Phys Act 20:149 38115056 10.1186/s12966-023-01542-x PMC10731871

[18] Moavero R, Di Micco V, Forte G, Voci A, Mazzone L, Valeriani M, Emberti Gialloreti L, Bruni O (2023) Screen exposure and sleep: how the COVID-19 pandemic influenced children and adolescents – a questionnaire-based study. Sleep Med 107:48–54 37116435 10.1016/j.sleep.2023.04.009 PMC10102534

[19] Moore KN, Do B, Wang SD, McAlister K, Chapman TM, Belcher BR, Dunton GF (2024) Long-term effects of the COVID-19 pandemic on children’s physical activity and sedentary behavior. Obes Sci Pract 10(1):e710 38263988 10.1002/osp4.710 PMC10804322

[20] Orgilés M, Delvecchio E, Francisco R, Mazzeschi C, Godinho C, Pedro M, Espada JP, Morales A (2024) Daily activities in European children and adolescents during COVID-19 school closure: a longitudinal study exploring physical activity, use of screens, and sleep patterns. J Prev 45(3):467–482 10.1007/s10935-024-00778-y 38564144

[21] Paruthi S, Brooks LJ, D’Ambrosio C, Hall WA, Kotagal S, Lloyd RM, Malow BA, Maski K, Nichols C, Quan SF, Rosen CL, Troester MM, Wise MS (2016) Recommended amount of sleep for pediatric populations: a consensus statement of the American Academy of Sleep Medicine. J Clin Sleep Med 12(6):785–786 27250809 10.5664/jcsm.5866 PMC4877308

[22] Portals-Riomao A, Nehari A, González-Gross M, Quesada-González C, Gesteiro E, Zapico AG (2025) The hidden effects of lockdown on child health: evidence from Madrid’s ASOMAD study. Sci 7(1):25. 10.3390/sci7010025

[23] Rodriguez-Ayllon M, Cadenas-Sánchez C, Estévez-López F, Muñoz NE, Mora-Gonzalez J, Migueles JH, Molina-García P, Henriksson H, Mena-Molina A, Martínez-Vizcaíno V, Catena A, Löf M, Erickson KI, Lubans DR, Ortega FB, Esteban-Cornejo I (2019) Role of physical activity and sedentary behavior in the mental health of preschoolers, children and adolescents: a systematic review and meta-analysis. Sports Med 49(9):1383–1410. 10.1007/s40279-019-01099-5 30993594 10.1007/s40279-019-01099-5

[24] Tatsiopoulou P, Holeva V, Nikopoulou VA, Parlapani E, Diakogiannis I (2024) Changes in sleep and association with screen exposure and diet among preschool children during the COVID-19 pandemic: a mixed methods study. J Child Fam Stud 33(2):395–406

[25] Tremblay MS, Carson V, Chaput JP, Connor Gorber S, Dinh T, Duggan M, Faulkner G, Gray CE, Gruber R, Janson K, Janssen I, Katzmarzyk PT, Kho ME, Latimer-Cheung AE, LeBlanc C, Okely AD, Olds T, Pate RR, Phillips A, Poitras VJ, Rodenburg S, Sampson M, Saunders TJ, Stone JA, Stratton G, Weiss SK, Zehr L (2016) Canadian 24-hour movement guidelines for children and youth: an integration of physical activity, sedentary behaviour, and sleep. Appl Physiol Nutr Metab 41(6 Suppl 3):S311–S327. 10.1139/apnm-2016-0151 27306437 10.1139/apnm-2016-0151

[26] Walker R, House D, Salway R, Emm-Collison L, Hollander LE, Sansum K, Breheny K, Churchward S, Williams JG, de Vocht F, Hollingworth W, Foster C, Jago R (2023) The new normal for children’s physical activity and screen viewing: a multi-perspective qualitative analysis of behaviours a year after the COVID-19 lockdowns in the UK. BMC Public Health 23(1):1432 37495976 10.1186/s12889-023-16021-y PMC10373375

[27] World Health Organization (2010) Recomendaciones mundiales sobre actividad física para la salud. https://iris.who.int/bitstream/handle/10665/44441/9789243599977_spa.pdf?sequence=1. Accessed 11 Sept 2025

[28] World Health Organization (2019) Guidelines on physical activity, sedentary behaviour and sleep for children under 5 years of age. https://iris.who.int/bitstream/handle/10665/311664/9789241550536-eng.pdf?sequence=1&isAllowed=y. Accessed 11 Sept 2025 31091057

[29] Zink J, Belcher BR, Imm K, Leventhal AM (2020) The relationship between screen-based sedentary behaviors and symptoms of depression and anxiety in youth: a systematic review of moderating variables. BMC Public Health 20:472. 10.1186/s12889-020-08572-1 32272906 10.1186/s12889-020-08572-1 PMC7147040

